# Mid- to late-life migraine diagnoses and risk of dementia: a national register-based follow-up study

**DOI:** 10.1186/s10194-020-01166-7

**Published:** 2020-08-06

**Authors:** Sabrina Islamoska, Åse Marie Hansen, Hui-Xin Wang, Anne Helene Garde, Per Kragh Andersen, Ellen Garde, Jakob Møller Hansen, Gunhild Waldemar, Kirsten Nabe-Nielsen

**Affiliations:** 1 Department of Public Health, University of Copenhagen, Øster Farimagsgade 5, 1014 Copenhagen, Denmark; 2 The National Research Centre for the Working Environment, Lersø Parkallé 105, 2100 Copenhagen, Denmark; 3 Stress Research Institute, Stockholm University, Frescati Hagväg 16A, 114 19 Stockholm, Sweden; 4 Danish Headache Center, Rigshospitalet – Glostrup, Valdemar Hansens Vej 5, 2600 Glostrup, Denmark; 5 Danish Headache Knowledge Center, Rigshospitalet – Glostrup, Valdemar Hansens Vej 5, 2600 Glostrup, Denmark; 6 Danish Dementia Research Centre, Rigshospitalet, University of Copenhagen, Section 6922, Juliane Mariesvej 28, 2100 Copenhagen, Denmark

**Keywords:** Migraine, Headache, Dementia, Neurology

## Abstract

**Background:**

Previous studies found an association between migraine and dementia, which are two leading causes of disability. However, these studies did not differentiate between migraine types and did not investigate all prevalent dementia subtypes. The main objective of this national register-based study was to investigate whether migraine was a risk factor for dementia. Additionally, we explored potential differences in dementia risk for migraine with and without aura.

**Methods:**

We obtained data on birth cohorts born between 1935 and 1956 (*n* = 1,657,890) from Danish national registers. Individuals registered with migraine before age 59 (*n* = 18,135) were matched (1:5) on sex and birthdate with individuals without migraine (*n* = 1,378,346). Migraine was defined by International Classification of Diseases (ICD) diagnoses and dementia was defined by ICD diagnoses and anti-dementia medication. After matching, 62,578 individuals were eligible for analysis. For the statistical analyses, we used Cox regression models and adjusted for socio-demographic factors and several psychiatric and somatic morbidities.

**Results:**

During a median follow-up time of 6.9 (IQR: 3.6–11.2) years, 207 individuals with migraine developed dementia. Compared with individuals without migraine, we found a 50% higher rate of dementia among individuals with migraine (HR = 1.50; 95% CI: 1.28–1.76). Individuals without aura had a 19% higher rate of dementia (HR = 1.19; 95% CI: 0.84–1.70), and individuals with aura had a two times higher rate of dementia (HR = 2.11; 95% CI: 1.48–3.00).

**Conclusions:**

Our findings support the hypothesis that migraine is a midlife risk factor for dementia in later life. The higher rate of dementia in individuals with a hospital-based diagnosis of migraine with aura emphasizes the need for studies on pathological mechanisms and potential preventative measures. Furthermore, given that only hospital-based migraine diagnoses were included in this study, future research should also investigate migraine cases derived from the primary healthcare system to include less severe migraine cases.

## Background

Migraine and dementia are among the most prevalent neurological disorders and leading causes of disability [1]. Whereas dementia is the most common neurological syndrome in older adults [1, 2], migraine is the most common neurological disorder across all ages [1, 3]. Previous epidemiological studies reported a positive association between migraine and Alzheimer’s disease (AD), vascular dementia (VaD) and unspecified dementia [4–8]. However, the majority of these studies are based on smaller populations and without a clear temporal separation of migraine and dementia [4, 5, 7, 8]. Other studies investigating the association between migraine and cognitive dysfunction have reported mixed results, which can be due to differences in study design, methodology and study population type and size [9]. The exact pathophysiological links between migraine and dementia are unknown, but mechanisms may include vascular disease and changes, increased amyloid plaque formation, inflammation, and deficits in nerve growth factors due to comorbid depression, increased cortisol levels due to psychological stress, brain structural changes in overlapping pain and memory networks [10], cardiovascular- and cerebrovascular events due to stroke and myocardial infarction [11], and structural brain abnormalities [12].

Migraine is a complex disorder characterized by episodes of severe, often unilateral throbbing or pulsating headache associated with multiple symptoms such as nausea, photophobia, and phonophobia [13]. Up to 1/3 of individuals with migraine experience aura characterized by reversible transient neurological symptoms, typically preceding the headache [14]. In particular, migraine with aura (MA) is observed to have a stronger association with structural brain abnormalities [12]. Yet, the specific effects of MA and migraine without aura (MO) in relation to a more comprehensive outcome measure of dementia have not been investigated.

Since brain changes indicative of cerebral small vessel disease increase the risk for most dementia types and cerebrovascular dysfunction [15], it is possible that the vascular component of migraine contribute to the risk of dementia [16]. Yet, due to the long preclinical phase of dementia [2, 17], the lack of substantial temporal separation of migraine and dementia in previous studies increases the risk of reverse causation, i.e., that early dementia-related vascular changes lead to migraine. To assess clinically relevant associations between migraine and dementia, longitudinal and population-based studies with larger samples are needed [18].

In this longitudinal population-based register study, the main objective was to investigate whether migraine registered in midlife was a risk factor for dementia in later life. Secondary objectives were to investigate whether MO and MA affected the risk of dementia, and to explore potential dose-response relations between migraine severity and dementia risk as well as differences across sex and educational level.

## Methods

### Study population and design

**Fig. 1:**
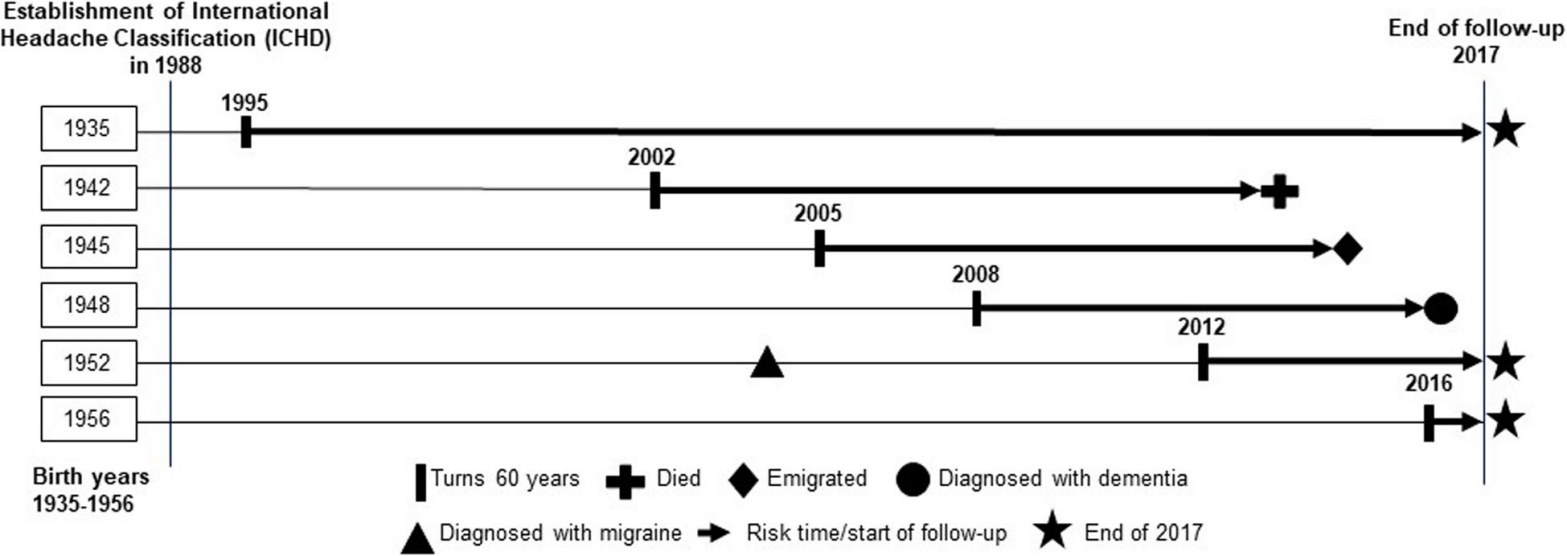
Study design. Birth cohorts born between 1935 and 1956 are illustrated from 1988 to 2017. Individuals were followed from when they turned 60 years until death, emigration, dementia diagnosis, or end of follow-up in 2017. Migraine diagnoses were registered from 1988 onwards. This figure illustrates the life course of a single individual from 6 of the 21 included birth cohorts exemplifying individuals turning 60 years and being followed in registers until endpoints

We used national register-based data on all inhabitants in Denmark of all ethnic origins born between 1935 and 1956 (*N* = 1,657,890), and the study covered 30 calendar years (1988–2017). Individuals registered with their first migraine diagnosis at age 31–58 were included. Individuals were considered at dementia risk at age ≥ 60 due to lower validity of dementia diagnoses in patients younger than 60 years [19, 20]. Because migraine diagnosis criteria was defined with the establishment of the International Classification of Headache Disorders (ICHD) in 1988 [21] and since incorporated in the International Classification of Diseases (ICD) [22], migraine information was included from 1988 to increase exposure assessment validity. We followed individuals in registers until being registered with dementia, death, emigration, or end of follow-up in 2017, whichever occurred first (Fig. 1).

**Fig. 2:**
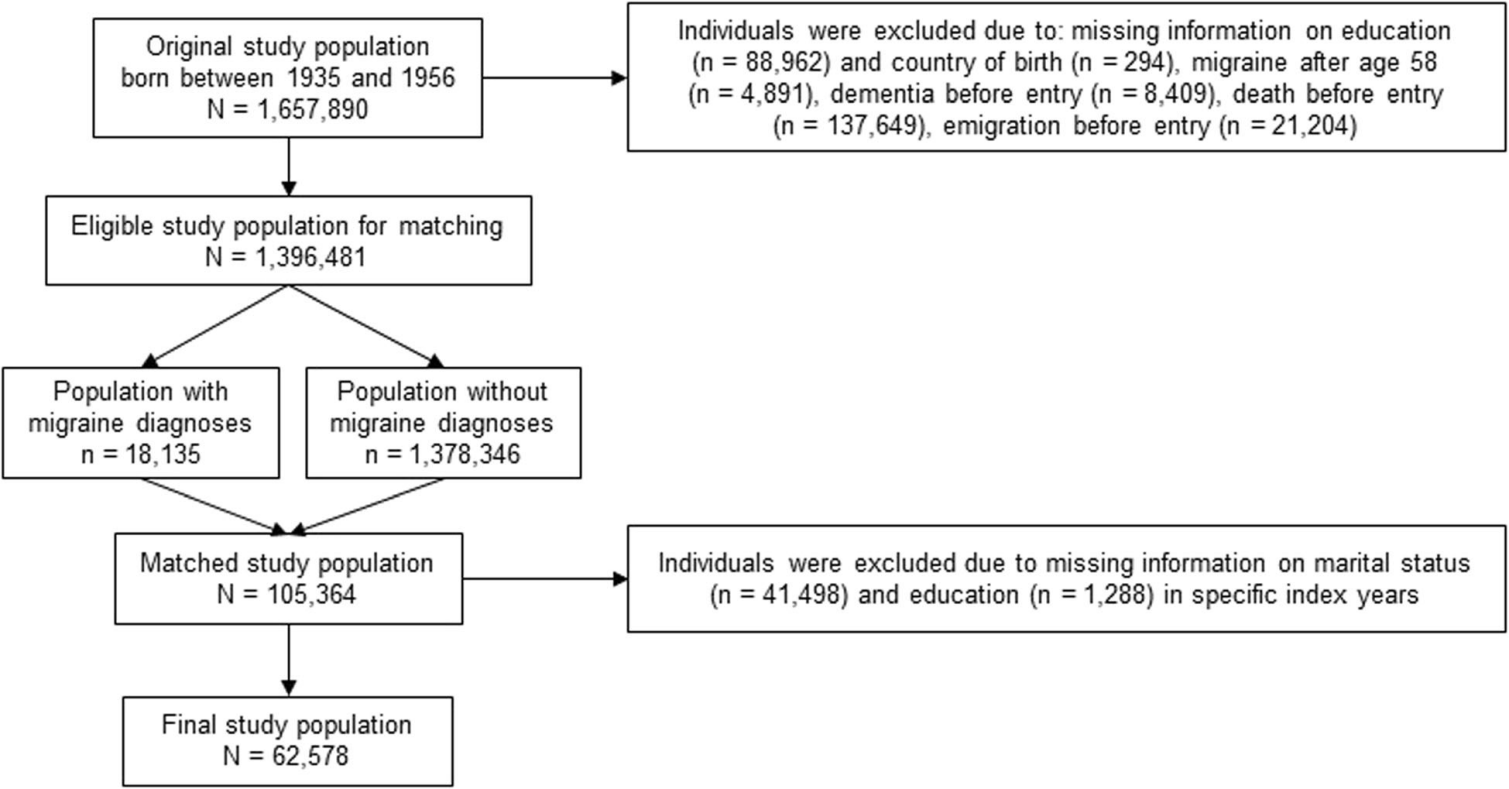
Flow chart of the study population selected for analyses (*N* = 62,578). Data were obtained on 1,657,890 inhabitants in Denmark born between 1935 and 1956. After excluding individuals due to missing information, migraine after age 58, or dementia, death or emigration before entry, 18,135 individuals with migraine diagnoses were matched with 1,378,346 individuals without migraine diagnoses. The matched study population consisted of 105,364 individuals, but also included individuals without information on education and marital status for specific index years and these individuals were therefore excluded after matching leaving 62,578 individuals eligible for analysis

Individuals were excluded if they died, emigrated or were registered with dementia before age 60, or due to missing information on covariates (Fig. 2). Lack of full coverage of register information on educational level and marital status [23] led to the exclusion of a substantial proportion of individuals, who were otherwise eligible for inclusion (*n* = 42,786; median age at index year: 35, IQR: 30–40; 70% women).

We used an exposure-matching procedure [24] based on sex and birthdate (± 30 days) to match sets of one individual with migraine with five individuals without migraine. All had to be alive on their 60 years’ birthday to be followed for incident dementia. In each matched set, we used the date of the migraine diagnosis to define an index year for all 6 individuals. This index date was used when retrieving information about covariates (see paragraph on covariates). The final study population included 62,578 individuals.

### Migraine

Migraine diagnoses were obtained from 1988 onwards from the Danish National Patient Register and the Danish Psychiatric Central Research Register, which include information on diagnoses registered by the secondary healthcare system, when an individual has been in contact with any hospital department due to inpatient, outpatient, or emergency room visits [25, 26]. Migraine was defined as first ever registration with one of the following migraine diagnoses: hemicrania ophthalmoplegica (ICD-8: 346.00), hemicrania alia definita (ICD-8: 346.08), hemicrania (ICD-8: 346.09); migraine (ICD-10: G43), MO (ICD-10: G43.0), MA (ICD-10: G43.1), status migrainosus (ICD-10: G43.2), complicated migraine (ICD-10: G43.3), other migraine (ICD-10: G43.8), or unspecified migraine (ICD-10: G43.9). First, we created one migraine variable defining individuals registered with any migraine diagnosis (none/any migraine diagnosis). Second, to investigate potential differences between MO and MA, we created a variable categorizing migraine into MO, MA and all other ICD-8 and ICD-10 migraine diagnoses (none/MO/MA/all other migraine diagnoses). Finally, we did an additional refinement of migraine to investigate migraine severity. We defined a migraine contact variable based on the total number of registered hospital contacts with migraine as primary diagnosis for contact before age 59 (range: 1–43 contacts; migraine cases: 8551; categories: 0/1/2/≥ 3 migraine contacts). Among individuals with migraine, we also investigated the rate of dementia when having more hospitals contacts due to other morbidities with migraine registered as secondary diagnosis at the same time (range: 1–51 contacts; migraine cases: 10,857). A hospital contact variable was defined identically to the migraine contact variable.

### Dementia

Dementia was defined as the first ever registration with dementia diagnosis or first redeemed anti-dementia medication, whichever came first. Data on dementia diagnoses were based on data from the Danish National Patient Register [25], Danish Psychiatric Central Research Register [26], and Danish Register of Causes of Death [27] by ICD codes from ICD-8 and ICD-10: unspecified dementia, AD, VaD, frontotemporal dementia, and Lewy body dementia (Table 1). In Denmark, national clinical guidelines for diagnosing dementia include standard diagnostic procedures, such as cognitive, neurological and mental assessment, neuroimaging, and laboratory tests [28]. Data on first redeemed prescriptions of anti-dementia medications were based on Anatomical Therapeutic Chemical (ATC) codes for cholinesterase-inhibitors of Donepezil (ATC: N06DA02), Rivastigmine (ATC: N06DA03), Galantamine (ATC: N06DA04), and the glutamate-receptor antagonist Memantine (ATC: N06DX01) obtained from the Danish National Prescription Registry [29]. Dementia patients in Denmark can receive a prescription for anti-dementia medication from all medical doctors, but only receive reimbursement when prescribed by a specialist in geriatrics, neurology or psychiatry [30]. Indications for using the glutamate-receptor antagonist Memantine in migraine treatment have been suggested [31], however, in Denmark, Memantine is only approved for dementia treatment [32].

**Table 1:** Diagnostic codes for dementia and included covariates from the International Classification of Diseases

Type of diagnoses	ICD-8 codes	ICD-10 codes
Dementia		
Alzheimer’s disease	290.10	F00.0–00.9, G30.0–30.9
Vascular dementia	293.09–19	F01.0–01.9
Frontotemporal dementia	290.11	F02.0
Lewy body dementia		G31.8
Unspecified and other dementia	290.09, 290.18–19	F03.9, G31.9
Headache		
Cephalalgia	791.99	DR51, DR519
Other headache syndromes		G44
Cluster headache syndrome		G44.0
Vascular headache		G44.1
Tension-type headache		G44.2
Chronic post-traumatic headache		G44.3
Drug-induced headache		G44.4
Other specified headache syndromes		G44.8
Psychiatric		
Schizophrenia, schizotypal and delusional disorders	295.09–99, 297.09–99	F20-F29
Mood affective disorders	296.09–99, 790.29	F30-F39
Other or unspecified psychoses	298.09–99, 299.00–09	
Neuroses	300.49–99	
Transient situational disturbances	307.99	
Acute stress reactions		F43.0
Post-traumatic stress disorders		F43.1
Adjustment disorders		F43.2
Other stress reactions		F43.8
Unspecified stress reactions		F43.9
Charlson Comorbidity Index		
Myocardial infarction	410	I21, I22, I23
Heart failure	427.09, 427.10, 427.11, 427.19, 428.99, 782.49	I50, I110, I130, I132
Peripheral vascular disease	440, 441, 442, 442, 444, 445	I70, I71, I72, I74, I77
Cerebral vascular accident	430–438	I60-I69, G45, G46
Pulmonary disease	490–493, 515–518	J40-J47, J60-J67, J684, J701, J703. J841, J920, J961, J982, J983
Connective tissue disorder	712, 716, 734, 446, 135.99	M05, M06, M08, M09, M30, M31, M32, M33, M34, M35, M36, D86
Ulcer disease	530.91, 530.98, 531–534	K221, K25-K28
Mild liver disease	571, 573.01, 573.04	B18, K700–703, K709, K71, K73, K74, K760
Diabetes	249.00, 249.06, 249.07, 249.09, 250.00, 250.06, 250.07, 250.09	E100, E101, E109, E110, E111, E119
Hemiplegia	334	G81, G82
Renal disease	403, 404, 580–584, 590.09, 593.19, 753.10, 753.20, 792	I12, I13, N00, N06, N07, N11, N14, N17-N19, Q61
Diabetes with complications	249.01–249.05, 249.08, 250.01–250.05, 250.08	E102-E108, E112-E118
Cancer	140–194	C00-C75
Leukemia	204–207	C91-C95
Lymphoma	200–203, 275.59	C81-C85, C88, C90, C96
Severe liver disease	070.00, 070.02, 070.04, 070.06, 070.08, 573.00, 456.00–456.10	B150, B160, B162, B190, K704, K72, K766, I85
Metastatic cancer	195–199	C76, C81
HIV	079.83	B21-B24

*ICD* International Classification of Diseases of the 8th (ICD-8) and 10th version (ICD-10)

### Covariates

Information on all covariates was extracted from national registers 1 year before the index year in order to ensure that these potential confounders were assessed before the exposure (i.e., migraine status). We included information on socio-demographic factors, such as birthdate, sex, country of origin (Denmark/Western countries/Non-Western countries) [33], marital status (unmarried/married), highest educational level [2] (low educational level defined by primary school/medium educational defined by upper secondary education, business high school, and vocational education and training/high educational level defined by short-term further education, middle-range education, bachelor’s degree, extended education, and research degree).

Headache diagnoses occurring before migraine diagnosis included: cephalalgia, other headache syndromes, cluster headache syndrome, vascular headache, tension-type headache, chronic post-traumatic headache, drug-induced headache, and other specified headache syndromes (Table 1).

We included information on psychiatric diagnoses occurring before migraine diagnosis as follows: schizophrenia, schizotypal and delusional disorders, mood affective disorders, psychoses, neuroses, and transient situational disturbances (Table 1).

To adjust for different morbidities potentially associated with migraine and dementia, we adjusted for multimorbidity defined by the Charlson Comorbidity Index (CCI) [34, 35]: myocardial infarction, heart failure, peripheral vascular disease, cerebrovascular disease, pulmonary disease, connective tissue disorder, peptic ulcer, liver disease, diabetes, diabetes complications, paraplegia, renal disease, cancer, metastatic cancer, severe liver disease, and human immunodeficiency virus (Table 1). Migraine may coexist with some of the included morbidities, or some morbidities could be consequences of migraine. In the present study, we treated morbidities as potential confounders, i.e., we adjusted for morbidities registered before the migraine diagnosis (or the index date for individuals without a migraine diagnosis). The purpose of including CCI was to adjust for multimorbidity, thereby, adjusting for some of the common risk factors for migraine and dementia, but also to take into account the health status of individuals with and without migraine.

All data in this study were obtained with approval from Statistics Denmark and the Danish Health Data Authority.

### Statistical analyses

Using frequency analyses, we investigated the distribution of socio-demographic factors, headache, psychiatric morbidities and CCI among individuals with and without migraine.

The Cox regression model was used to investigate the association between migraine and dementia. We calculated hazard rate ratios (HR) of dementia and used age as the time scale beginning at age 60. As we used data from individuals born across a wide year range and the probability of exposure misclassification depends on birth cohort, we stratified analyses on birth cohort to ensure that comparisons were made among individuals with the same probability of misclassification of exposure.

First, we investigated the association between any migraine diagnosis and dementia by using an overall migraine variable including all migraine diagnoses. We adjusted for potential confounders using two models: Model 1 included sex; and Model 2 included sex, country of origin, marital status, educational level, headache, psychiatric morbidities, and CCI. Second, we investigated the associations of MO and MA with dementia and adjusted for covariates in Models 1 and 2. Furthermore, we investigated migraine severity by using the migraine contact variable as exposure in relation to dementia adjusting for covariates in Models 1 and 2. Similarly, we also investigated the association between several hospital contacts among individuals with migraine and dementia. Individuals without migraine were the reference group in all analyses.

We also examined interactions between migraine and sex and educational level, respectively, on the rate of dementia by adding interaction terms to the Cox regression model.

To ensure the temporal relation between migraine and dementia, we strictly separated the timing of migraine and dementia by performing sensitivity analyses in which we postponed the start of follow-up with at least 5, 10, 15, and 20 years after the index date. This was done in order to reduce the risk of reverse causation, i.e., that the risk of migraine was affected by underlying cerebrovascular mechanisms of dementia, since dementia can have a long preclinical phase starting already in midlife [17].

The Cox regression model assumes that the ratio of hazards for any two groups is constant over time [36], therefore, the proportionality of hazards was tested. By using a Cox regression model including covariates of Model 2, we tested the significance of a time dependent interaction between time and covariates [37]. We considered *p*-values < 0.05 as statistically significant.

We used SAS Enterprise Guide version 7.1 in combination with SAS 9.4 to conduct all analyses.

## Results

In this national register-based follow-up study, 18,135 were registered with migraine and of those, 10,857 were eligible for inclusion. After matching, the study population included 62,578 individuals (Fig. 1). Median age at index year was 49 years, and approximately 70% were women. The cohort with any migraine did not differ substantially from the comparison cohort (Table 2).

**Table 2:** Baseline characteristics of the study population based on migraine diagnoses shown in prevalence and medians (*N* = 62,578)

	Any ICD-8 and ICD-10 migraine diagnosis	Comparison cohort	Any ICD-8 migraine diagnosis	Any ICD-10 migraine diagnosis	Migraine without aura in ICD-10	Migraine with aura in ICD-10
Characteristics	***n*** = 10,857 (17%)	***n*** = 51,721 (83%)	***n*** = 2183 (20%)	***n*** = 8674 (80%)	***n*** = 2845 (26%)	***n*** = 1698 (16%)
**Age, median (IQR)**						
Years	49 (44–53)	49 (44–53)	43 (38–47)	50 (46–54)	50 (46–54)	51 (47–54)
**Sex, No. (%)**						
Women	7975 (73)	38,104 (74)	1574 (72)	6401 (74)	2239 (79)	1200 (71)
**Country of origin, No. (%)**						
Danish	10,087 (93)	49,177 (95)	2118 (97)	7970 (92)	2615 (92)	1586 (93)
Western	272 (2)	1399 (3)	35 (2)	237 (3)	76 (3)	55 (3)
Non-Western	498 (5)	1145 (2)	31 (1)	467 (5)	154 (5)	57 (3)
**Marital status, No. (%)**						
Unmarried	3029 (28)	15,027 (29)	566 (26)	2,463 (28)	824 (29)	432 (25)
**Educational level, No. (%)**						
Low	3799 (35)	17,757 (34)	976 (45)	2,823 (32)	904 (32)	530 (31)
Medium	4173 (38)	20,366 (40)	814 (37)	3359 (39)	1089 (38)	656 (39)
High	2885 (27)	13,598 (26)	394 (18)	2492 (29)	852 (30)	512 (30)
**Previous headache diagnoses, No. (%)**						
Yes	600 (6)	524 (1)	63 (3)	537 (6)	210 (7)	86 (5)
**Previous psychiatric diagnoses, No. (%)**						
Yes	748 (7)	2185 (4)	126 (6)	622 (7)	191 (7)	115 (7)
**Charlson Comorbidity Index, No. (%)**						
0	10,059 (93)	48,729 (94)	2135 (98)	7925 (91)	2,577 (91)	1542 (91)
≥1	789 (7)	2992 (6)	49 (2)	749 (9)	268 (9)	156 (9)
**Migraine contacts, median (IQR)**						
Number	1 (1–2)	–	1 (1–1)	1 (1–2)	1 (1–2)	1 (1–2)

*ICD* International Classification of Diseases 8th (ICD-8) and 10th version (ICD-10), *IQR* interquartile ranges

During a median follow-up time of 6.9 years (interquartile range (IQR): 3.6–11.2) from age 60 years, 207 individuals with migraine were identified as dementia cases at a median age of 68 years (IQR: 64–73). Among individuals without migraine, 640 individuals had dementia at a median age of 69 years (IQR: 65–73). The median time between registered migraine and dementia was 18.3 years (IQR: 13.4–22.7) and the total follow-up time was 30 years from 1988 to 2017. Individuals were primarily registered with dementia in patient data (82%), then prescription data [17], and mortality data (1%).

Individuals with migraine had a 1.50 times higher rate of dementia (95% CI: 1.28–1.76). Individuals with MO had a 1.19 times higher rate of dementia (95% CI: 0.84–1.70), and individuals with MA had a 2.11 times higher rate (95% CI: 1.48–3.00). There was a significant difference between the rates of dementia for MO and MA (*p* = 0.02). All other migraine types, excluding MO and MA, were associated with a 1.48 times higher rate of dementia (95% CI: 1.23–1.78). We found a 1.38 times higher rate of dementia for individuals with one migraine contact (95% CI: 1.13–1.69), and a 1.51 times higher rate when having two contacts (95% CI: 1.01–2.27) compared with individuals without migraine. Among individuals with migraine, the rate of dementia was 1.49 times higher (95% CI: 1.25–1.77) when having one hospital contact, and 1.80 times higher (95% CI 1.29–2.51) with two hospital contacts. Due to a low number of dementia cases among individuals with ≥ 3 migraine and hospital contacts, these results are probably not reliable (Table 3).

**Table 3:** Hazard rate ratios of dementia associated with migraine diagnoses (*N* = 62,578)

Analysis	Migraine diagnosis	No. of individuals	Minimum follow-up years between migraine and dementia	Dementia cases/ person-years	Model 1 HR (95% CI)	Model 2 HR (95% CI)
Main analysis	None	51,721	≥ 1	640/402,006	1.00	1.00
	Any	10,857	≥ 1	207/83,926	1.55 (1.32–1.81)	1.50 (1.28–1.76)
	Migraine without aura	2845	≥ 1	34/19,995	1.21 (0.85–1.72)	1.19 (0.84–1.70)
	Migraine with aura	1698	≥ 1	33/11,604	2.13 (1.50–3.03)	2.11 (1.48–3.00)
	All other migraine types	6314	≥ 1	140/52,328	1.55 (1.29–1.86)	1.48 (1.23–1.78)
Sensitivity analysis	Any	10,773	≥ 5	205/81,326	1.55 (1.32–1.81)	1.50 (1.28–1.76)
	Any	10,198	≥ 10	185/74,055	1.49 (1.26–1.75)	1.43 (1.21–1.69)
	Any	9677	≥ 15	174/69,486	1.53 (1.29–1.82)	1.48 (1.25–1.76)
	Any	9271	≥ 20	164/67,344	1.57 (1.32–1.87)	1.53 (1.28–1.83)
**Additional refinement of exposure**	**Number of contacts**					
Migraine contacts^a^	0	40,718	≥ 1	499/313,832	1.00	1.00
	1	6233	≥ 1	121/50,101	1.44 (1.18–1.76)	1.38 (1.13–1.69)
	2	1428	≥ 1	25/10,432	1.63 (1.09–2.43)	1.51 (1.01–2.27)
	≥ 3	890	≥ 1	6/5438	0.94 (0.42–2.11)	0.89 (0.40–2.00)
Hospital contacts^b^	0	51,721	≥ 1	640/402,006	1.00	1.00
	1	7836	≥ 1	162/63,184	1.53 (1.29–1.82)	1.49 (1.25–1.77)
	2	1810	≥ 1	37/13,305	1.89 (1.36–2.64)	1.80 (1.29–2.51)
	≥ 3	1211	≥ 1	8/7437	0.92 (0.46–1.85)	0.89 (0.44–1.79)

Model 1: adjusted for sex. Model 2: adjusted for sex, country of origin, marital status, educational level, headache diagnoses, psychiatric morbidities and Charlson Comorbidity Index (CCI)

*HR* Hazard rate ratios, *95% CI* 95% confidence intervals

^a^ Analyses of migraine contacts and dementia were based on individuals with any migraine diagnosis registered as the primary reason for hospital contact (*N* = 49,269)

^b^ Analyses of hospital contacts and dementia were based on individuals with any migraine diagnosis registered as either primary or secondary reason for hospital contact (*N* = 62,578)

Sensitivity analyses showed that the magnitude and direction of the association between having any migraine, MO, MA and other types of migraine and dementia remained when including different time intervals between migraine and start of follow-up (at least 5, 10, 15 and 20 years between index date and start of follow-up) (Table 3).

Interaction analyses showed a slightly higher rate of dementia in women with migraine than in men with migraine, and a higher rate was also observed in individuals with a higher educational level than in other educational levels. However, these differences were not statistically significant (Table 4).

**Table 4:** Hazard rate ratios of dementia associated with migraine diagnoses grouped by sex or educational level (*N* = 62,578)

Characteristics	No. of individuals	Dementia cases/person-years	HR (95% CI)	***P*** value for interaction
**Sex**				0.6^a^
Men				
Without migraine	13,617	186/102,687	1.00	
With migraine	2882	57/21,913	1.39 (1.03–1.88)	
Women				
Without migraine	38,104	454/299,319	1.00	
With migraine	7975	150/62,013	1.54 (1.28–1.86)	
**Educational level**				0.8^b^
Low				
Without migraine	17,757	292/147,296	1.00	
With migraine	3799	92/31,028	1.47 (1.16–1.87)	
Medium				
Without migraine	20,366	229/159,620	1.00	
With migraine	4173	72/32,484	1.45 (1.11–1.89)	
High				
Without migraine	13,598	119/95,090	1.00	
With migraine	2885	43/20,414	1.64 (1.16–2.33)	

In all models, any migraine diagnosis was investigated as exposure. All models were adjusted for sex, all other migraine diagnoses, country of origin, marital status, educational level, other headache diagnoses, psychiatric morbidities and Charlson Comorbidity Index

*HR* Hazard rate ratios, *95% CI* 95% confidence intervals

^a^ Interaction tested between migraine and sex

^b^ Interaction tested between migraine and educational levels

The assumption of proportional hazards could not be rejected, thus, the overall rate of dementia for any migraine did not vary significantly with time after age 60.

## Discussion

### Main results

We observed a higher rate of dementia after age 60 among individuals with migraine in midlife compared with individuals without migraine. MA was associated with the highest rate of dementia. The rate of dementia was higher among individuals with more hospital registrations of migraine. The observed associations did not change substantially when taking several confounders into account, or including longer time between registration of migraine and dementia.

### Comparison with previous research

Our findings are comparable with other studies reporting an association between both self-reported [7, 8] and clinically diagnosed migraine [4–6] and a higher risk of dementia. In addition, our findings are also supported by previously suggested plausible pathological mechanisms between migraine and dementia [10].

Among individuals with migraine, a three times higher risk has been reported for VaD [8] and all-cause dementia [7], and a four times higher risk of AD [7]. In one of these studies, individuals were on average 75 years when reporting migraine history [7], thus, reverse causation can be an issue. Other studies did not find an association between migraine and cognitive decline [18] or dementia [38]. These disparities could potentially be due to methodological characteristics, e.g. the inclusion of individuals reporting migraine at an older age, using too short follow-up, including self-reported migraine, recalling migraine history when having impaired cognitive function, and possible difficulties in identifying small changes in cognitive functioning despite the use of standardized measurement methods [18]. A review concluded that small sample sizes and limited information on migraine characteristics, such as attack frequency, could be reasons for not finding an association between migraine and cognitive decline [18], which could also be the explanation for not observing an association between migraine and dementia [38]. Migraine attacks have shown to be associated with poor cognitive performance compared with headache-free periods [9]. However, different studies have shown conflicting results regarding the association between migraine and risk of cognitive decline [9], and it is debatable whether migraine-related cognitive complaints during attacks would qualify for a diagnosis of mild cognitive impairment.

Other studies using migraine based on ICD-9 and ICD-10 found similar or somewhat lower dementia risks compared with our study [4–6]. Two studies considered individuals at dementia risk after age 60 [4, 5], however, one of these studies retrieved information on migraine and dementia simultaneously in the age range 60–80 years [4]. This yields a potential overestimation of the association between migraine and dementia due to the risk of reverse causation or shared underlying causes. We accounted for potential reverse causation by strictly separating timing of exposure from outcome and only included migraine cases before age 59 and dementia cases after age 60. Sensitivity analyses were also conducted to assess the degree of bias due to reverse causation.

Compared with MO, MA is associated with a higher risk of structural brain abnormalities and cardio- and cerebrovascular events [11, 12]. In our study, individuals with MA had the highest rate of dementia, which is in line with previous research supporting potentially stronger vascular mechanisms in MA [11, 12]. Our findings add to the knowledge on severe long-term consequences of especially MA. To fully assess the impact of MO and MA and later brain health, potentially modifying factors as attack frequency and treatment should be included.

### Strengths and limitations

This is the first national register-based study investigating hospital-based migraine diagnoses in midlife and dementia risk in later life. Information on covariates dated back to 1958, migraine dated back to 1988 and follow-up lasted until 2017 with a low risk of loss to follow-up. Compared with other studies using register-based migraine information, we included all ICD-8 and ICD-10 diagnoses of migraine. We took into account potential confounding of other headache diagnoses as well as several different morbidities prior to migraine diagnosis. Still, the results did not change substantially. Based on the large population size, we were able to use an exposure-matching procedure and match one individual with migraine with five individuals without migraine. We examined migraine severity and our findings highlighted the importance of monitoring severe migraine.

Based on national recommendations by the Danish Health Authority, uncomplicated migraine cases should be treated by their own general practitioner, while complicated, unsolved or rare migraine cases should be treated by specialized neurologists or headache units [39]. As we only included migraine cases treated in hospital settings, we assume that the less severe and well-managed migraine cases treated by general practitioners are likely to be misclassified as non-exposed in our study. This may lead to an underestimation of the migraine-dementia association. In addition, the general migraine prevalence is 16% in Denmark [40], while the prevalence in our study was 1.3% (18,135 hospital-based migraine cases out of 1,396,481 individuals), which also yields a potential underestimation of the actual dementia risk. Still, including only severe hospital-diagnosed migraine cases may overestimate the observed association between migraine and dementia. Hospital-based migraine diagnoses may be associated with more complications, e.g. migraine chronification or medication overuse. Compared with episodic migraine, individuals with chronic migraine are observed to have more cognitive impairment [41]. Furthermore, a review found that individuals with complicated medication overuse headache (MOH) have better outcomes after inpatient treatment indicating that cases of MOH may be inclined to seek hospital treatment [42]. This suggest that migraine cases with hospital-based diagnoses may include more severe or complicated cases. Thus, our findings of a higher dementia risk are only generalizable to individuals with severe migraine requiring hospital contact and do not necessarily apply to less severe migraine cases.

Some research suggests that migraine is a progressive disorder due to being associated with later cardio- and cerebrovascular events [11]. However, since migraine may as well remit over time [3], we cannot preclude that the included migraine cases may comprise individuals with remitted migraine, which can lead to an underestimation of the migraine-dementia association. We acknowledge that there is individual variation in patients’ burden of migraine and, thereby, also later vascular brain changes, which may depend on disease duration, attack frequency, and type of migraine [12].

Another limitation is that age at first hospital contact with migraine may not correspond with age at disease onset. Migraine is most prevalent in the age group 25 to 55 years [3], while the median age of individuals when registered with a migraine diagnosis was 49 years in our population. Based on diagnostic criteria of migraine, migraine is defined as a primary headache, which is not caused by another disorder [13]. However, migraine is a complex disorder and especially for MA, the diagnostic challenges when discriminating between strokes, syncope, seizure and MA [43] may delay the diagnosis and thereby the time of registration in hospital data. Furthermore, the completeness of migraine ascertainment in the National Patient Register may have changed over time, since outpatient data were only included from 1995, and the inclusion of data from private hospitals were not obligatory until 2003 [25]. In addition, when restricting our population to migraine cases registered from 1988 onwards in order to increase the validity of migraine diagnoses, we also excluded individuals, who might have been registered with migraine before age 31.

Our results are strengthened by using validated dementia diagnoses from patient registers [44]. Yet, 60% of dementia patients are not diagnosed [45], therefore, register data underestimate the dementia prevalence. For a more comprehensive detection of individuals with dementia, both register-based dementia diagnoses and prescriptions were included. However, we cannot rule out that individuals with more hospital contacts (e.g., due to migraine) are more likely to be registered with dementia, even if their true dementia risk is not higher. This detection bias would yield an overestimation of the association between migraine and dementia, which was partly addressed by adjusting for multimorbidity. However, the registration of morbidity diagnoses in registers may not reflect the actual onset of disease, as they may have existed for a longer time before actual diagnosis. Furthermore, despite adjusting for numerous confounders, dementia risk might be influenced by lifestyle risk factors, medication, or other neurological disorders [3]. In addition, since the number of dementia cases was low particularly among individuals with ≥ 3 migraine or hospital contacts including only 6 and 8 dementia cases respectively, this limits the validity of these specific analyses.

The oldest individuals were 82 years old at end of follow-up. Therefore, including older individuals could lead to different results, since dementia is an age-related syndrome with typical onset starting from 80 to 89 years [46]. Thus, our study population is at lower risk of dementia diagnosis and death in general.

Some anti-migraine medications as ergotamines and triptans have vasoactive actions [47]. Previous research showed that triptans were not associated with ischemic complications and vascular events [48, 49]. Treatment with ergot alkaloids in migraine cases has not been verified as a stroke risk factor, but high ergotamine consumption is possibly associated with risk of serious ischemic complications [48]. Current evidence does not suggest that the most used anti-migraine medication, triptans, increase the risk of cardiovascular events [47]. Therefore, anti-migraine medication does not seem to be a potential pathway to dementia. In terms of non-steroidal anti-inflammatory drugs (NSAIDs), which are also used in migraine treatment [50], a meta-analysis reported that individuals using NSAIDs seemed to have a lower risk of AD [51]. Yet, a review concluded that there were no significant benefits of NSAIDs in the treatment of AD [52], thus, the literature is inconclusive regarding NSAID and its effect on dementia.

## Conclusions

In conclusion, our results support the hypothesis that migraine in midlife is a risk factor for dementia in later life, especially for individuals with MA. It is conceivable that some traits in certain individuals with migraine may entail a more pronounced dementia risk. Frequent hospital visits resulted in a higher rate of dementia. However, given the limitations of this study, we cannot conclude on the dementia risk among individuals with migraine treated outside hospitals. Future research should preferably include individuals with migraine registered in both the primary and secondary healthcare system to be able to investigate mild, moderate and severe migraines and dementia risk. Our findings emphasize the need for studies on the pathophysiology linking migraine with dementia in order to identify preventive measures.

## Data Availability

The data that support the findings of this study are available from Statistics Denmark and the Danish Health Data Authority, but restrictions apply to the availability of these data, which were used under license for the current study, and so are not publicly available.

## Abbreviations

AD: Alzheimer’s disease
95% CI: 95% confidence interval
HR: Hazard rate ratio
ICD: International Classification of Diseases
ICHD: International Classification of Headache Disorders
MA: Migraine with aura
MO: Migraine without aura
VaD: Vascular dementia
